# Zinc Potentiates the Renoprotective Effects of SGLT2 Inhibitors in Experimental Diabetes Mellitus in Rats

**DOI:** 10.3390/life16050793

**Published:** 2026-05-09

**Authors:** Irina Claudia Anton, Carmen Solcan, Liliana Mititelu Tartau, Cornelia Amalinei, Mihaela Poroch, Vladimir Poroch, Beatrice Rozalina Buca, Cosmin-Gabriel Tartau, Ana-Maria Pelin, Gina Eosefina Botnariu

**Affiliations:** 1 Faculty of Medicine, Grigore T. Popa University of Medicine and Pharmacy, Universitatii St. 16, 700115 Iasi, Romania; irinaanton@taktfest.ro (I.C.A.); liliana.tartau@umfiasi.ro (L.M.T.); cornelia.amalinei@umfiasi.ro (C.A.); boanca.mihaela@umfiasi.ro (M.P.); vladimir.poroch@umfiasi.ro (V.P.); beatrice-rozalina.buca@umfiasi.ro (B.R.B.); cosmin.tartau@gmail.com (C.-G.T.); ginabotnariu66@gmail.com (G.E.B.); 2 Faculty of Veterinary Medicine, Ion Ionescu de la Brad University of Life Sciences, Aleea Mihail Sadoveanu No. 3, 700490 Iasi, Romania; 3 Department of Morphological and Functional Sciences, Dunarea de Jos University, 800010 Galati, Romania; anapelin@gmail.com

**Keywords:** diabetes, rats, SGLT2 inhibitors, empagliflozin, zinc, renal protection

## Abstract

Background: Diabetic kidney disease is a common and serious complication of type 2 diabetes mellitus (T2DM) and represents a major contributor to chronic kidney disease (CKD) globally. While sodium-glucose cotransporter-2 (SGLT2) inhibitors have demonstrated significant renoprotective effects, the potential advantages of combining these agents with micronutrients such as zinc (Zn), known for its antioxidant, anti-inflammatory, and metabolic regulatory properties, have not been fully investigated. This study aimed to assess the effects of dapagliflozin (DAPA) and empagliflozin (EMPA), administered either alone or alongside Zn, in an experimental diabetes model. Methods: T2DM was induced in Sprague-Dawley rats through a high-fat diet (HFD) followed by a low dose of streptozotocin (STZ). Seven experimental groups were established: a control group, an untreated diabetic group, and treatment groups receiving DAPA, EMPA, or their combinations with Zn. Metabolic parameters, renal function, and histopathological alterations were assessed, while immunohistochemistry was used to evaluate the expression of inflammatory and fibrotic markers. Results: Diabetic rats exhibited sustained hyperglycemia, metabolic imbalance, and significant renal damage, accompanied by elevated levels of inflammatory and fibrotic markers. Treatment with SGLT2 inhibitors improved metabolic status, mitigated kidney injury, and reduced inflammatory marker expression. Zn association further potentiated these effects, with the most pronounced benefits observed when combined with EMPA. Conclusions: These findings suggest that SGLT2 inhibitors exert strong renoprotective effects in experimental diabetic nephropathy. Zn supplementation may amplify these benefits through its antioxidant and anti-inflammatory actions. The combination of EMPA and Zn demonstrated the greatest protective effect, highlighting the potential of multi-target therapeutic strategies in diabetic kidney disease.

## 1. Introduction

The prevalence of type 2 diabetes mellitus (T2DM) has increased globally over recent decades, making it a major public health concern worldwide. Current epidemiological estimates indicate that more than 500 million adults are affected by T2DM, and this number is projected to rise substantially in the coming decades due to population aging, sedentary lifestyles, and the growing prevalence of obesity [1,2]. Chronic hyperglycemia in T2DM arises from both peripheral insulin resistance and a gradual decline in pancreatic β-cell function. Persistent metabolic imbalance promotes the development of multiple microvascular and macrovascular complications that considerably contribute to increased morbidity and mortality among diabetic patients [3].

Diabetic kidney disease (DKD) is among the primary causes of chronic kidney disease (CKD) and end-stage renal disease (ESRD) globally [4,5]. DKD develops through multiple interacting mechanisms, including metabolic, hemodynamic, oxidative, and inflammatory processes [6]. Sustained hyperglycemia activates several pathogenic pathways, including the formation of advanced glycation end products, mitochondrial dysfunction, increased production of reactive oxygen species, and activation of pro-inflammatory transcription factors. These mechanisms contribute to progressive renal injury through endothelial dysfunction, cellular apoptosis, and extracellular matrix accumulation.

From a histopathological perspective, diabetic nephropathy is characterized by structural alterations affecting both glomerular and tubulointerstitial compartments. Typical changes include glomerular basement membrane thickening, mesangial matrix expansion, podocyte injury, and progressive tubulointerstitial fibrosis [7]. Renal fibrosis represents a central event in the progression of DKD and is primarily driven by the activation of myofibroblasts and excessive deposition of extracellular matrix components [8]. Several molecular markers have been associated with these pathological processes. Alpha-smooth muscle actin (α-SMA) is widely recognized as a marker of myofibroblast activation and fibrotic remodeling [8], while nuclear factor-kappa B (NF-κB) functions as a key transcription factor regulating inflammatory signaling pathways activated by hyperglycemia and oxidative stress [9].

Inflammation is increasingly recognized as a major contributor to diabetic renal injury. Elevated glucose levels promote the release of pro-inflammatory mediators such as cytokines and chemokines, as well as adhesion molecules [9]. Among these mediators, intercellular adhesion molecule-1 (ICAM-1) and vascular cell adhesion molecule-1 (VCAM-1) play important roles in vascular inflammation and endothelial dysfunction associated with diabetic nephropathy [10]. In parallel, alterations in epithelial cell adhesion and integrity contribute to structural remodeling of renal tissue. N-cadherin, a key adhesion molecule involved in maintaining epithelial architecture and intercellular communication, has been associated with renal injury and epithelial–mesenchymal transition (EMT), a process that promotes fibrotic remodeling and progression of kidney disease.

In recent years, SGLT2 inhibitors have emerged as an important therapeutic class for the management of T2DM with demonstrated benefits beyond glycemic control. By preventing glucose reuptake in the proximal tubules of the kidney, these compounds enhance glucosuria and contribute to better metabolic regulation [11]. In addition to their glucose-lowering effect, SGLT2 inhibitors exert multiple kidney protective actions, including reductions in intraglomerular pressure, improvement of renal hemodynamics, reduction in oxidative processes and regulation of inflammatory signaling pathways [12]. Large-scale clinical research has indicated that, in people with T2DM and CKD, SGLT2 inhibitors are associated with a meaningful reduction in both the progression of renal impairment and the incidence of cardiovascular events [13,14]. Among these agents, dapagliflozin (DAPA) and empagliflozin (EMPA) have shown substantial renal protective effects, including reductions in albuminuria, a slower decline in glomerular filtration rate and an improvement in kidney tissue damage [15,16,17].

Despite these therapeutic advances, the complex and multifactorial pathophysiology of diabetic nephropathy suggests that strategies targeting multiple pathogenic mechanisms simultaneously may provide enhanced protection against renal injury. In this setting, micronutrients that exhibit antioxidant and anti-inflammatory effects have attracted growing interest as possible supportive treatment options. Zn is a vital trace element that participates in a wide range of biological functions, such as insulin production, storage, and release, along with the control of oxidative stress and inflammatory activity [18,19,20]. Zn deficiency has been associated with impaired glucose metabolism, increased oxidative stress, enhanced inflammatory signaling, and altered lipid metabolism, all of which are implicated in the onset and progression of diabetes-related complications [18,21,,22,23]. Experimental studies have suggested that Zn supplementation may improve glycemic control, reduce oxidative damage, and attenuate inflammatory responses under diabetic conditions [24,25].

Animal models play a critical role in elucidating the mechanisms underlying diabetic complications and in evaluating potential therapeutic interventions. The combination of a HFD with low-dose STZ administration represents a commonly used experimental model that mimics important metabolic characteristics of human T2DM, such as insulin resistance, partial impairment of β-cell function, and sustained high blood glucose levels [26,27]. Such models allow the comprehensive assessment of metabolic disturbances, renal functional parameters, and histopathological alterations associated with the development of diabetic nephropathy [17,28,29].

Although the renal protective effects of SGLT2 inhibitors have been extensively demonstrated, the potential benefits of combining these agents with micronutrients capable of modulating oxidative stress and inflammation remain insufficiently investigated. In particular, the interaction between SGLT2 inhibitors and Zn supplementation has received limited attention in experimental models of diabetic nephropathy. Understanding whether Zn may enhance the beneficial effects of SGLT2 inhibitors could provide valuable insights into novel adjunctive therapeutic strategies targeting the multiple pathogenic pathways involved in DKD progression.

This study was designed to investigate the effects of DAPA and EMPA, used alone or together with Zn supplementation, in a STZ-induced rat model of T2DM. By integrating biochemical, histological, and immunohistochemical analyses of key inflammatory and fibrotic markers, including NF-κB, ICAM-1, VCAM-1, α-SMA, and N-cadherin, this study investigates whether Zn supplementation can potentiate the kidney protective effects of SGLT2 inhibitors and attenuate diabetes-induced renal injury. The comparative evaluation of two clinically relevant SGLT2 inhibitors in combination with Zn may provide new insights into potential synergistic therapeutic strategies targeting metabolic dysregulation, inflammation, and fibrotic remodeling in diabetic kidney disease.

## 2. Materials and Methods

### 2.1. Substances

DAPA and EMPA were used in their marketed formulations to ensure clinical relevance of the dosing regimen. DAPA tablets (10 mg, Forxiga^®^) were obtained from AstraZeneca (Södertälje, Sweden), and EMPA tablets (25 mg, Jardiance^®^) were obtained from Boehringer Ingelheim International GmbH (Ingelheim am Rhein, Germany). The following chemicals used in the study, including STZ (~75% β-anomer, ≥98% by HPLC, MW 265.22, catalogue code S0130), citrate buffer (catalogue code C2488, solution 0.09 M), zinc chloride (MW 136.3, catalogue code 208086), and distilled water (MW 18.02, catalogue code 07-6061), were obtained from Sigma-Aldrich (Steinheim, Germany; www.sigma-aldrich.com, accessed 21 February 2026).

### 2.2. Animals

A total of 35 male Sprague-Dawley rats (8–10 weeks old, 200–250 g) were used in this study. The animals were sourced from the Cantacuzino National Medical-Military Research and Development Institute in Băneasa, Bucharest, Romania, and kept under standard housing conditions, including a controlled 12 h light/dark cycle, a constant temperature of 22 ± 2 °C, and ad libitum access to water. Only male animals were included in the study because sex is known to significantly influence diabetes development in experimental models; specifically, male pancreatic β-cells are more susceptible to STZ-induced cytotoxic effects than those of females, due to the protective effects of estrogens on β-cell function [30]. The Sprague-Dawley strain was selected due to its greater tendency to develop T2DM-related metabolic disturbances in experimental settings, compared to other strains, such as Wistar rats, a susceptibility attributed to strain-dependent genetic background, and increased sensitivity to diet-induced obesity and insulin resistance [31].

### 2.3. Experimental Protocol

**Figure 1:**
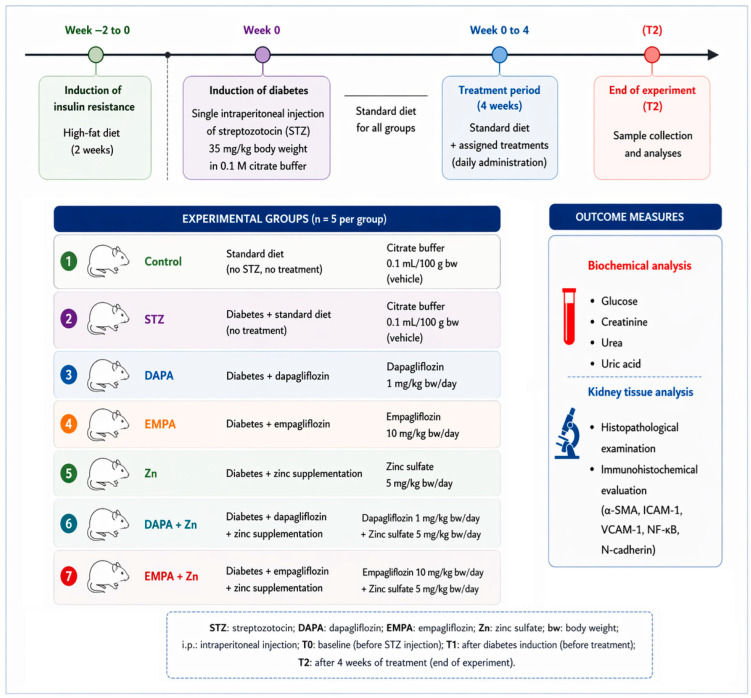
The flow diagram illustrating the experimental protocol.

The model consisting of the combination of a HFD and low-dose STZ administration is considered an alternative experimental approach for inducing of T2DM (Figure 1). The pathogenesis of diabetes in this animal model has been shown to be the closest to that described in humans with T2DM. Thus, insulin resistance and compensatory hyperinsulinemia have been shown be key pathophysiological features with strong predictive value for the development of T2DM in both clinical and experimental models of disease [32,33]. The experiments were carried out under controlled laboratory conditions at the CEMEX—Advanced Research and Development Center for Experimental Medicine ‘Ostin C. Mungiu’ of the Grigore T. Popa University of Medicine and Pharmacy, Iași, with standardized environmental parameters and consistent animal care maintained throughout the study.

#### 2.3.1. Animal Model of T2DM

The animals received a high-fat diet (formulated for this experiment in collaboration with the Cantacuzino Institute Bucharest), containing 40% carbohydrates, 15% protein, and 45% fat, administered over a 2-week period at a daily ration of 25 g per rat. The ingredients used in preparing the HFD were: corn, wheat, barley, soybean meal, sunflower meal, monocalcium phosphate, methionine, calcium carbonate, salt, premix for mice and rats, full-fat soy, lard, and sugar. After the two-week administration of the HFD and until the completion of the experiment, the animals were fed a standard diet in an amount of 25 g/rat/day. The control group was fed a standard laboratory chow diet (normal diet) containing approximately 10–15% of calories from fat, 50–60% from carbohydrates, and 20–25% from protein. The diet was provided ad libitum and was matched in micronutrient content to the high-fat diet, differing primarily in fat content. At the beginning of the experiment, after the two weeks of HFD, and at the end of the experiment, the animals were weighed to record the body weight gain.

At the end of the 2 weeks of HFD, the rats were subjected to a 12 h fasting period, and blood glucose levels were assessed with the aid of a portable glucometer (Accu-Chek Activa, Roche, Basel, Belgium). Afterwards, the animals received a single intraperitoneal injection of 35 mg/kg body weight STZ, dissolved in 0.1 M citrate buffer, pH 4.5. The STZ solution was freshly prepared and injected within 5 min of dissolution, as its stability in citrate buffer is 15 to 20 min after preparation. Treatment duration was 4 weeks post-T2DM confirmation. Three days after STZ administration, blood glucose was re-evaluated to confirm the onset of T2DM. Rats showing blood glucose values above 250 mg/dL were classified as diabetic and selected for inclusion in the study.

The prepared solutions were administered orally (using an esogastric device) for 4 weeks, as follows: 1 mg/kg body weight/day DAPA, 10 mg/kg body weight/day EMPA, 5 mg/kg body weight/day zinc chloride, 1 mg/kg body weight/day DAPA + 5 mg/kg body weight/day zinc chloride, 10 mg/kg body weight/day EMPA + 5 mg/kg body weight/day zinc chloride, and +5 mg/kg body weight/day zinc chloride. The control group (representing the negative control) consisted of non-diabetic animals and received 0.1 mL/100 g body weight of citrate buffer (0.1 M), whereas the STZ-coded group (serving as the positive control in the experiment) received 35 mg/kg body weight STZ dissolved in 0.1 M citrate buffer (Figure 1). At the end of the treatment period, blood glucose was measured again using a glucometer in order to record the changes according to the treatment received. The values recorded in the control group were considered reference values.

#### 2.3.2. Blood Analysis

Blood samples for laboratory analyses were obtained from one of the lateral tail veins. For this procedure, the animals were placed in a restraint device to ensure adequate immobilization and to minimize stress during sampling. To promote venous dilation, the tail was immersed in water heated to 42 °C for approximately 40 s. Subsequently, one of the lateral veins was identified and disinfected using 2% chlorhexidine. Venipuncture was performed with a fine peripheral venous catheter equipped with a stylet, inserted obliquely along the course of the vessel, about 5 cm away from the tail tip [34,35]. Using a syringe attached to the catheter, approximately 0.3 mL of blood was drawn from each animal, and the samples obtained were subsequently used for the analysis of biochemical parameters, including glucose, creatinine, ureea and uric acid levels. Laboratory investigations were conducted at three predefined time points during the experimental period: T0, corresponding to the beginning of the experiment and representing the baseline evaluation; T1, performed after two weeks; and T2, carried out at the end of the experiment (Figure 1).

#### 2.3.3. Statistical Processing of Data

Results are expressed as mean ± standard deviation (SD). Statistical analysis was carried out using SPSS software (version 17.0, IBM, New York, NY, USA). One-way ANOVA followed by Tukey’s post hoc test was applied to compare groups. A *p*-value below 0.05 was regarded as statistically significant. Given the small sample size (n = 5 per group), effect sizes (Cohen’s d) were calculated, allowing robust statistical evaluation while adhering to the 3Rs principle (Replacement, Reduction, Refinement) to ensure ethical use of animals.

Because of the relatively small sample size, a post hoc power analysis was performed to evaluate the study’s capacity to identify meaningful biological differences. The calculated power, based on effect sizes previously described in STZ-induced diabetes models, exceeded 80% for large effects (Cohen’s d > 1.2) at α = 0.05, supporting the adequacy of the sample size for detecting substantial biochemical, histological, and immunohistochemical changes.

Effect sizes (Cohen’s d) were calculated to complement hypothesis testing and to provide a quantitative measure of biological relevance. Several comparisons yielded very large effect sizes, reflecting low within-group variability and pronounced between-group differences, typical of controlled preclinical studies. Integrating significance testing with effect size evaluation allowed a robust and comprehensive interpretation of the experimental outcomes while maintaining ethical standards in animal research.

#### 2.3.4. Histopathological Evaluation

Upon completion of the experiment, blood samples were harvested from the animals in each group for biochemical analyses, as well as tissue samples for the histopathological examination of the kidney fragments (Figure 1). The specimens were collected immediately after euthanasia and placed in Bouin’s fixative solution for 24 h. They were then dehydrated in increasing concentrations of ethyl alcohol (70%, 75%, 80%, 85%, 90%, 96%, and absolute ethanol), for 3 h in each bath.

The next step was the clearing stage, performed in three xylene baths, one hour in each bath. Subsequently, the specimens were placed in three successive paraffin baths at 60 °C in a thermostat. After paraffin embedding, tissue sections were prepared at a thickness of 5 μm and then subjected to hematoxylin and eosin (H&E) staining along with immunohistochemical (IHC) analysis. H&E staining procedure was carried out using the following sequence of steps: deparaffinization in xylene, hydration in alcohol, staining with hematoxylin followed by eosin, dehydration in alcohol, clearing in xylene, and mounting with Canada balsam. IHC staining was carried out using specific antibody markers: VCAM-1 antibody (M/K-2): sc-18864; ICAM-1 antibody (G-5) SC-8439 (mouse); α-SMA (MA5-11547) (mouse); NF-κB (AA 143–158 ABIN2627580) (rabbit); N-cadherin (NB 120-11512) (mouse); anti-insulin ABI6939789 (mouse).

The slides were deparaffinized and placed in a microwave oven for 10 min at 95 °C in an acidic citrate buffer (10 mmol, pH 6). Following a 20 min cooling period, the samples were rinsed twice with phosphate-buffered saline (PBS) for 5 min each and subsequently left overnight at 4 °C in a humidified chamber with primary antibody incubation.

The primary antibodies used included VCAM-1 (diluted 1:500), ICAM-1 (1:200), α-SMA (1:800), NF-κB (1:100), N-cadherin (1:500), and anti-insulin for mice (1:100). The following day, the slides were washed three times in PBS for 5 min and incubated with secondary antibodies, including Goat Anti-Rabbit HRP (ab205718) diluted 1:100 for NF-κB and the Goat Anti-Mouse Leica kit for VCAM-1, ICAM-1, N-cadherin, and anti-insulin. Detection was achieved using 3,3′-diaminobenzidine (DAB) as the chromogen, after which the slides were counterstained with hematoxylin.

A quantitative analysis of the primary antibody immunolabelling was performed using QuPath software (version 0.5.1, Edinburgh, UK). The preparation of three sections for each animal in the group was followed by the capture of digital images at 400× magnification from five different fields within each section. In these fields, positive cells were automatically counted, and a final mean was calculated per group. This was expressed as the number of positive cells per mm^2^.

### 2.4. Research Ethics

Animal studies were conducted following approval by the University Ethics Committee (Ethical Approval No. 30/14 January 2021; Authorization from the Veterinary Public Health Directorate No. 30/24 February 2021).

At the end of the experimental period, animals were euthanized using inhalational anesthesia with isoflurane. Animals were placed in an induction chamber and exposed to 3% isoflurane in oxygen until loss of consciousness was achieved. Depth of anesthesia was assessed by the absence of righting reflex and pedal withdrawal reflex. Throughout the procedure, animals were continuously monitored for respiratory pattern, heart rate (assessed visually or by palpation), and general responsiveness. Isoflurane exposure was maintained for at least 5 min following the loss of consciousness to ensure deep anesthesia. Animals were evaluated at regular intervals (every 1–2 min) for progressive depression of vital functions, including reduced respiratory rate and complete loss of reflex activity. Death was confirmed by the absence of spontaneous respiration, lack of cardiac activity, and fixed, dilated pupils.

All procedures were conducted in accordance with international and national guidelines for the care and use of laboratory animals, with all efforts made to minimize animal suffering [36,37].

## 3. Results

### 3.1. Variation in Mean Body Weight

When considering all three time points, body weight increased progressively from T0 to T2 in all groups, with the most pronounced overall increase observed in the STZ group.

**Figure 2:**
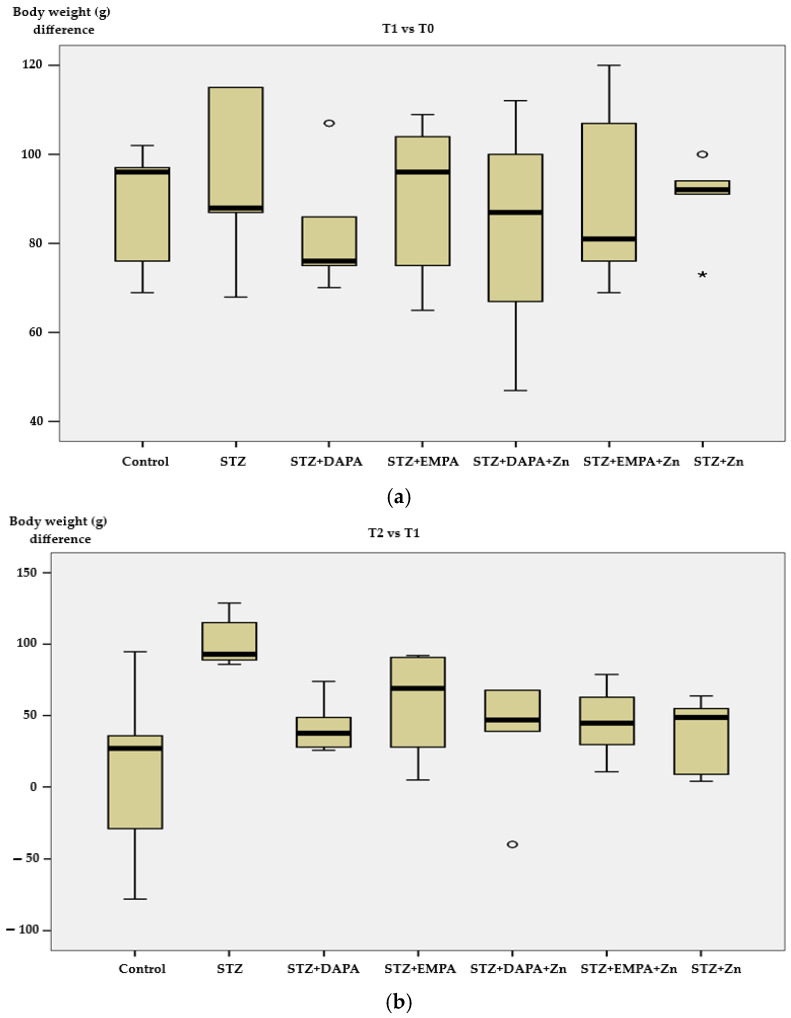
Changes in body weight across experimental groups. (**a**) Body weight variation between T1 and T0; (**b**) body weight variation between T2 and T1. T0 represents baseline (prior to diabetes induction), T1 corresponds to 2 weeks after high-fat diet and diabetes induction, and T2 represents the end of the experimental period (after 4 weeks of treatment). Data are expressed as mean ± SD (n = 5 animals per group). STZ: streptozotocin; DAPA: dapagliflozin; EMPA: empagliflozin. The circles represent the significance <0.01 and the asterisks <0.001.

At baseline (T0), body weights varied between 222 and 261 g, without significant differences in mean values across the experimental groups (*p* = 0.090). The lowest average body weight was observed in the STZ + EMPA + Zn group (231 ± 8.63 g), whereas the highest mean value was recorded in the STZ + DAPA + Zn group (247.80 ± 9.52 g) (Figure 2a). At time point T1, the mean body weight increased in all analyzed groups; however, these differences were not statistically significant (*p* = 0.947). The smallest increase was observed in the STZ + DAPA + Zn-treated group (82.60 g ± 25.97), while the greatest weight gain occurred in the STZ group (94.60 g ± 20.26) (Figure 2a).

Individual changes in body weight at T2 showed considerable variability, ranging from a decrease of 78 g to an increase of 129 g. However, the mean body weight increased in all analyzed groups, reaching statistical significance (*p* = 0.031). The greatest increase was once more recorded in the group receiving STZ treatment (102.40 g ± 18.73), with individual values ranging between 86 g and 126 g (Figure 2b).

### 3.2. Variation in Blood Glucose Levels

When considering all three time points, blood glucose levels increased markedly from T0 to T1 following diabetes induction and remained elevated at T2 in the untreated STZ group. In contrast, treated groups exhibited a more moderate increase from T0 to T1 and a partial reduction or stabilization at T2, suggesting improved glycemic control, particularly in animals receiving SGLT2 inhibitors.

**Figure 3:**
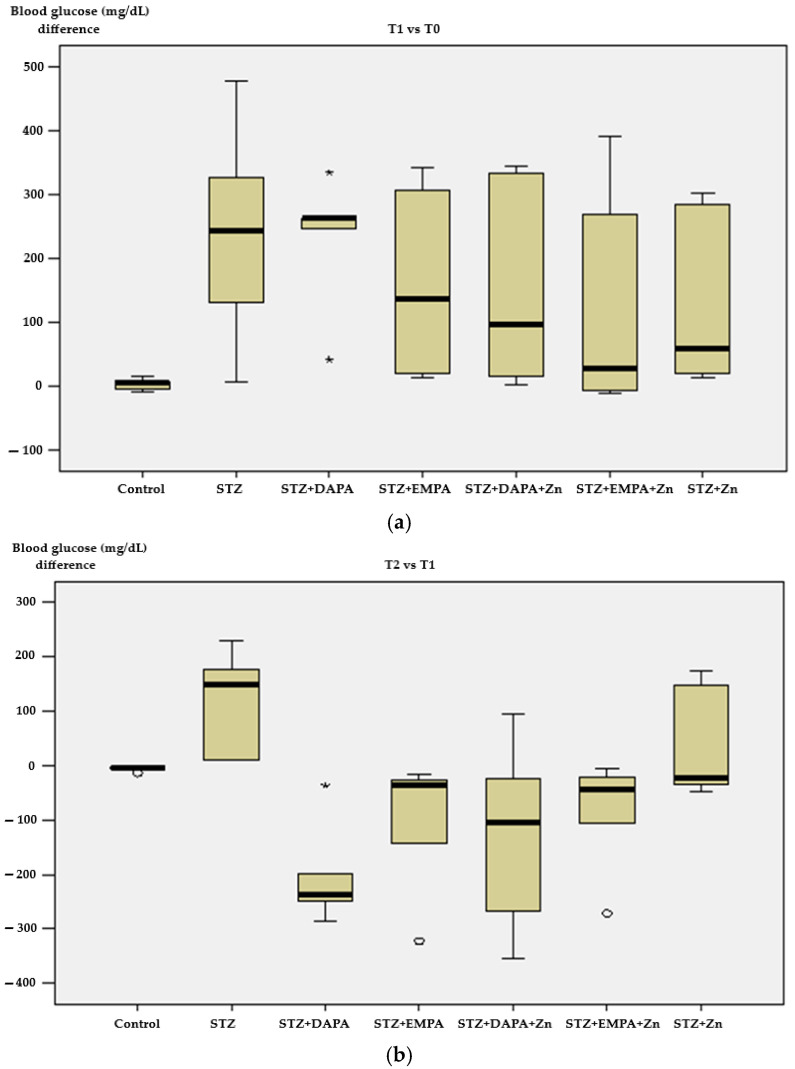
Changes in blood glucose levels across experimental groups. (**a**) Blood glucose variation between T1 and T0; (**b**) blood glucose variation between T2 and T1. T0 represents baseline (prior to diabetes induction), T1 corresponds to 2 weeks after high-fat diet and diabetes induction, and T2 represents the end of the experimental period (after 4 weeks of treatment). Data are expressed as mean ± SD (n = 5 animals per group). STZ: streptozotocin; DAPA: dapagliflozin; EMPA: empagliflozin.

Blood glucose measurements at T0 showed values ranging from 94 to 141 mg/dL, with no statistically significant differences in mean levels across the experimental groups (*p* = 0.306). The lowest average concentrations were recorded in the Control group (115.20 ± 5.36 mg/dL) as well as in the STZ + DAPA group (115.60 ± 6.43 mg/dL), whereas the highest mean value was observed in the STZ + EMPA + Zn group (129.20 ± 8.04 mg/dL) (Figure 3a). At T1, blood glucose levels varied widely, from 112 mg/dL to 589 mg/dL, and the comparison of group means showed noteworthy differences between the treated groups and the Control group (*p* = 0.032). The Control group exhibited the lowest mean level (118.20 mg/dL ± 6.06), while the STZ group showed the highest mean value (359.60 mg/dL ± 175.85) (Figure 3a). By T2, glucose levels ranged from 98 mg/dL to 600 mg/dL, and the analysis of mean values across the study groups indicated statistically significant differences (*p* = 0.029). The Control group continued to show the lowest average value (113.20 ± 2.17 mg/dL), while the STZ group again exhibited the highest mean concentration (475.20 ± 191.55 mg/dL) (Figure 3b).

In the Control group, greater differences in body weight were, in more than 61% of cases, accompanied by larger changes in blood glucose between T2 and T1 (r = +0.611; *p* = 0.274). In contrast, the STZ group showed an inverse pattern, where over 54% of increased weight differences were associated with reduced changes in glycaemia over the same period (r = −0.541; *p* = 0.347). In the STZ + Zn group, the change in blood glucose levels showed a notable inverse correlation with weight difference, indicating that greater increases in body weight were linked to more limited variations between glycemic values at T2 and T1 (r = −0.975; *p* = 0.011) (Figure 3b). In both the STZ + DAPA (r = +0.444; *p* = 0.453) and STZ + DAPA + Zn (r = +0.589; *p* = 0.296) groups, variations in blood glucose levels were moderately and positively correlated with corresponding changes in body weight from T1 to T2. Similarly, in the STZ + EMPA (r = +0.254; *p* = 0.681) and STZ + EMPA + Zn (r = +0.612; *p* = 0.273) groups, glycemic changes were directly correlated with weight differences, although the strength of the association ranged from low to moderate (Figure 3b).

### 3.3. Variation of Creatinine, Urea and Uric Acid Levels in Blood

No relevant variations were observed in the serum levels of creatinine, urea, and uric acid between time points T1 and T0, indicating that these biochemical parameters remained stable during the initial half of the experimental period.

To improve comparability, changes between T0 and T2 were assessed across all groups. STZ-induced diabetic animals showed increased creatinine, urea, and uric acid levels at T2 compared to T0, whereas treated groups, particularly those receiving SGLT2 inhibitors in combination with Zn, exhibited attenuated changes, with the smallest differences observed in the STZ + EMPA + Zn group, suggesting a stronger renoprotective effect.

**Figure 4:**
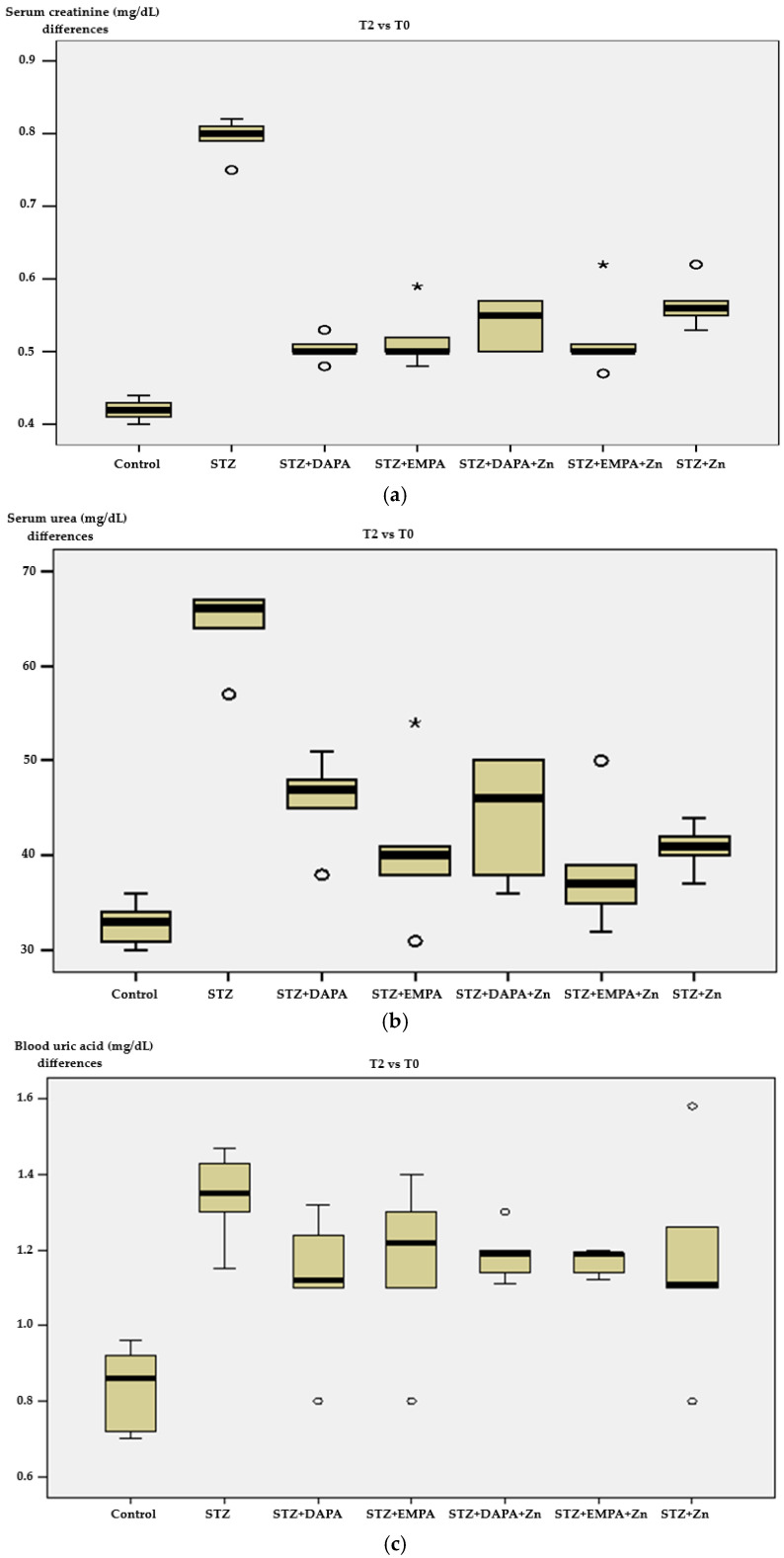
Changes in renal biochemical parameters across experimental groups. (**a**) Serum creatinine, (**b**) urea, and (**c**) uric acid levels between T2 and T0. T0 represents baseline (prior to diabetes induction), and T2 corresponds to the end of the experimental period (after 4 weeks of treatment). Data are expressed as mean ± SD (n = 5 animals per group). STZ: streptozotocin; DAPA: dapagliflozin; EMPA: empagliflozin.

Serum creatinine values ranged from 0.47 mg/dL to 0.75 mg/dL, showing statistically significant variation in mean levels among the experimental groups (*p* = 0.001) (Figure 4a). The lowest mean value was recorded in the Control group (0.42 mg/dL ± 0.02), while the highest mean level was observed in the STZ group (0.79 mg/dL ± 0.03) indicating impaired renal function following diabetes induction.

Treatment with SGLT2 inhibitors partially attenuated this increase. The STZ + DAPA group showed the lowest creatinine level among the treated groups (0.50 ± 0.02 mg/dL), followed by STZ + DAPA + Zn (0.51 ± 0.03 mg/dL) and STZ + EMPA/STZ + EMPA + Zn (both 0.52 mg/dL). The STZ + Zn group presented a slightly higher mean value (0.57 ± 0.03 mg/dL), suggesting a weaker renal protective effect when Zn was administered alone (Figure 3a). Effect size analysis confirmed the magnitude of the differences in serum creatinine levels among the experimental groups. The comparison between the control and STZ groups showed an extremely large effect size (Cohen’s d ≈ 14.8), reflecting the strong impact of STZ-induced diabetes on renal function. All treatment groups showed very large reductions in serum creatinine levels compared with the STZ group, with Cohen’s d values ranging from 7.1 to 10.6, and the largest effect size observed for the STZ vs. STZ + DAPA + Zn comparison (d ≈ 10.6), indicating a strong renal protective effect of this association (Supplementary Materials Table S1).

Serum urea levels ranged between 30 and 105 mg/dL, with notable differences detected between the experimental groups (*p* = 0.001). The control group showed the lowest average level (32.80 ± 2.39 mg/dL), while the highest mean concentration was recorded in the STZ group (71.80 ± 18.97 mg/dL), confirming the presence of diabetes-associated renal impairment (Figure 4b). Within the treated cohorts, the STZ + DAPA group showed the highest average urea concentration (45.80 ± 4.87 mg/dL), although values remained markedly lower than in the untreated STZ group. The other treatment groups displayed intermediate values, suggesting partial improvement in renal function following pharmacological intervention (Figure 4b).

Effect size analysis confirmed the magnitude of the differences in serum urea levels between experimental groups. The comparison between the control and STZ groups yielded an substantial large effect size (Cohen’s d = 2.89), suggesting a strong impact of STZ-induced diabetes on renal function. Comparisons between the STZ group and treated groups showed very large effect sizes, ranging from 1.67 to 1.93, suggesting a substantial reduction in serum urea levels following treatment. The largest effect sizes were observed for STZ vs. STZ + Zn (d ≈ 1.93) and STZ vs. STZ + DAPA + Zn (d ≈ 1.90) (Supplementary Materials Table S2).

Uric acid values spanned from 0.70 to 1.58 mg/dL across the experimental groups. Statistically significant differences in average levels were observed exclusively when comparing the Control and STZ groups (*p* = 0.006) (Figure 4c). The Control group registered the lowest mean concentration (0.83 ± 0.12 mg/dL), whereas the STZ group demonstrated the highest average value (1.34 ± 0.13 mg/dL), suggesting an increase in purine metabolism associated with diabetes. Among the treated groups, uric acid levels showed moderate increases compared with controls, with the highest mean value observed in the STZ + DAPA + Zn group (1.19 ± 0.07 mg/dL).

The comparison between the control and STZ groups revealed a large-magnitude effect size (Cohen’s d ≈ 4.06), reflecting a strong metabolic alteration associated with STZ-induced diabetes. Comparisons between the STZ group and treated groups showed small to moderate effect sizes, ranging from 0.74 to 1.72, but these differences did not reach statistical relevance. The largest effect size was observed for the STZ vs. STZ + EMPA + Zn comparison (d ≈ 1.72), suggesting a potential influence of combined therapy on uric acid metabolism (Supplementary Materials Table S3).

Based on the calculated effect sizes and a sample size of five animals per group (α = 0.05), the estimated statistical power exceeded 80% for the majority of comparisons, indicating that the sample size used in this study was sufficient to detect biologically meaningful differences in kidney function indices. All these indicate that STZ-induced diabetes results in serious renal dysfunction, reflected by increased serum creatinine, urea, and uric acid levels. The use of SGLT2 inhibitors, particularly DAPA, resulted in a partial improvement of renal function markers.

### 3.4. Histopathological Findings

**Figure 5:**
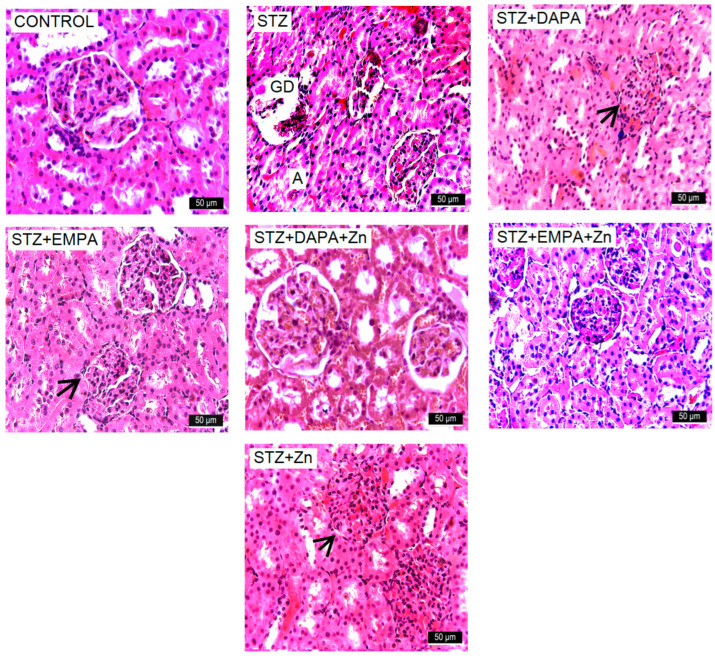
Representative images of the kidneys structure in rats with STZ-induced T2DM. The renal morphology appears to be normal in the control group. In contrast, the STZ-exposed group exhibits glomerular damage (GD), degeneration and necrosis of epithelial cells, along side tubular dilation and atrophy (A). The kidneys of rats administered with DAPA or EMPA displayed occasional glomeruli that were closely associated with the Bowman capsule (black arrow), characterized by reduced or absent capsule space. In the experimental groups receiving DAPA or EMPA along with Zn, the lesions were less severe, with affected regions alternating with areas exhibiting normal STZ + EMPA + Zn morphology, which showed the least changes. Original magnification ×400. H&E stain.

Following the evaluation of kidney histological specimens, normal renal morphology was observed in the Control group. In the STZ-exposed group, structural damage was identified, including glomerular deterioration, degeneration and fibrosis, epithelial cell degeneration and necrosis, along with dilation and atrophy of the renal tubules (Figure 5). Localized inflammatory cell infiltration was also observed within the intertubular regions of this group.

A progressive reduction in the severity of these lesions was observed across the experimental groups, following the sequence below: STZ + Zn, DAPA + STZ, DAPA + STZ + Zn, EMPA + STZ, and EMPA + STZ + Zn. In renal tissue from rats treated with DAPA or EMPA, isolated instances of glomeruli adherent to Bowman’s capsule were identified, often associated with a reduced or even absent capsular space. Furthermore, mild hydropic changes and limited areas of tubular necrosis were observed, but these alterations were relatively sparse and less extensive compared with untreated conditions. In animals from the SGLT-2 inhibitor and Zn groups, the histological lesions were noticeably reduced in severity, with areas of damage interspersed with regions exhibiting preserved, normal morphology (Figure 5). Examination of H&E-stained kidney sections showed that the most pronounced morphological changes occurred in the STZ group, affecting both the Malpighian corpuscles and the renal tubules. In the treated groups, lesion severity progressively declined from the STZ + Zn group to the STZ + EMPA + Zn group, which exhibited the mildest alterations (Figure 5).

**Figure 6:**
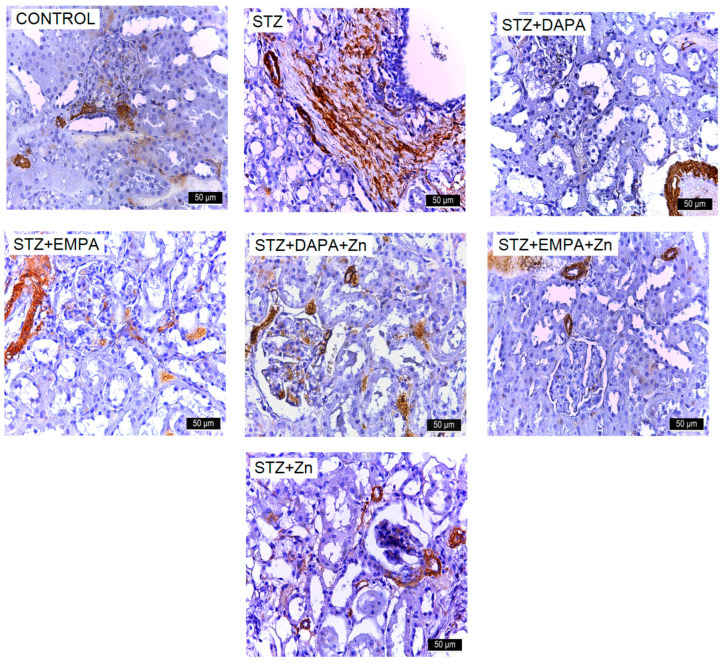
Representative immunohistochemical images of α-SMA expression in kidney tissue of rats with STZ-induced T2DM. Kidney sections were collected from the cortical region at the end of the experimental period (T2). Images are representative of findings observed in all animals from each experimental group (n = 5 per group). Increased immunoreactivity for α-SMA is observed in the STZ group. Treatment with DAPA and EMPA, alone or in combination with Zn, reduced marker expression, with the most pronounced effect observed in the EMPA + Zn group. All images are presented at the same magnification (×400), and scale bars represent 50 μm. STZ: streptozotocin; DAPA: dapagliflozin; EMPA: empagliflozin.

Elevated renal glucose levels stimulated the expression of mesenchymal markers, including α-SMA, while downregulating N-cadherin, indicating that high-glucose conditions promote EMT in proximal tubular cells. These phenotypic changes were accompanied by increased extracellular matrix production, reflected in the upregulation of mesenchymal proteins such as α-SMA. α-SMA positivity was present in the smooth muscle cells of renal arterioles in the Control group and in the majority of experimental groups. However, in the STZ and STZ + Zn groups, α-SMA expression extended beyond the arterioles, being also identified in cortical regions and within glomerular structures. Treatment with EMPA and DAPA attenuated the expression of this marker (Figure 6).

**Figure 7:**
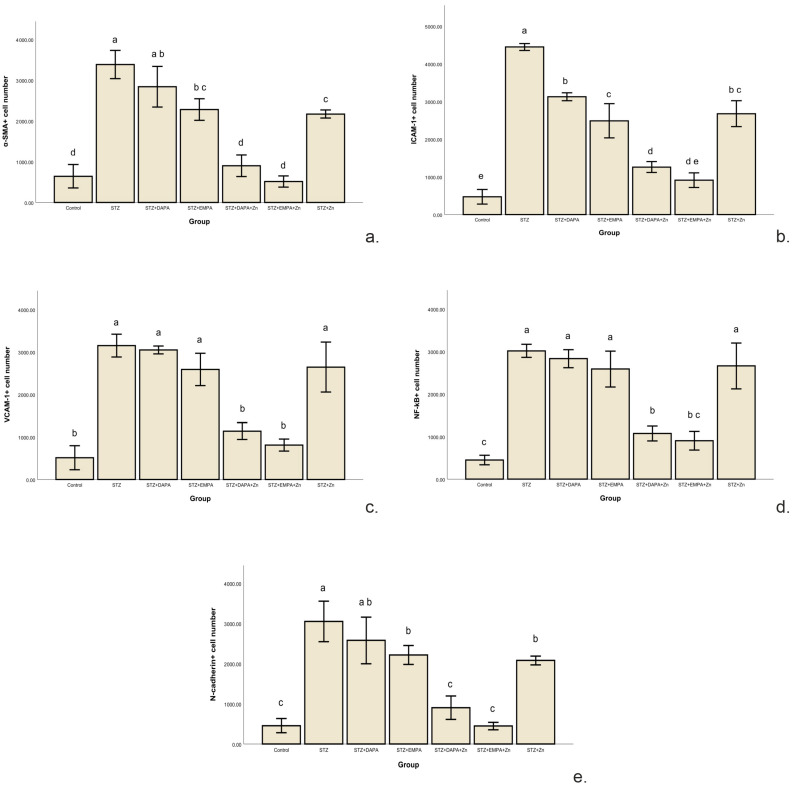
Changes in renal immunohistochemical markers across experimental groups. (**a**) Number of α-SMA-positive cells per mm^2^, (**b**) Number of ICAM-1-positive cells per mm^2^, (**c**) Number of VCAM-1-positive cells per mm^2^, (**d**) Number of NF-κB-positive cells per mm^2^, (**e**) Number of N-cadherin-positive cells per mm^2^. Data are expressed as mean ± SD (n = 5 animals per group). ^a,b,c,d^ The presence of different superscripts across groups indicates significant differences (*p* < 0.05) based on Tukey’s HSD test. STZ: streptozotocin; DAPA: dapagliflozin; EMPA: empagliflozin.

The number of α-SMA-positive cells ranged from 512.48 ± 136.65 to 3385 ± 346.93, with statistically significant differences between the group means (F(6, 28) = 72.97, *p* < 0.001). The STZ group (3385.02 positive cells/mm^2^ ± 346.93) exhibited higher number of α-SMA positive cells (*p* < 0.001) compared to the Control group (642.19 positive cells/mm^2^ ± 286.27). Treatment with SGLT2 inhibitors attenuated this increase. The STZ + EMPA + Zn group showed the lowest level of α-SMA immunostaining among the treatment groups, with no significant differences compared to the STZ + DAPA + Zn group (*p* = 0.04) and the Control group (*p* = 0.81). The STZ + EMPA group had a higher number of α-SMA-positive cells, with no significant difference compared to the STZ + Zn group (*p* = 0.99) (Figure 7a). Effect size analysis confirmed the magnitude of the differences in α-SMA immunostaining levels among the experimental groups. The comparison between the control and STZ groups showed an extremely large effect size (Cohen’s d ≈ 8.6), reflecting the strong impact of STZ-induced diabetes on renal function. All treatment groups showed very large reductions in α-SMA immunostaining levels compared with the STZ group, with Cohen’s d values ranging from 1.27 to 10.89, and the largest effect size observed for the STZ vs. STZ + EMPA + Zn comparison (d ≈ 10.89), indicating a strong renal protective effect of this association.

**Figure 8:**
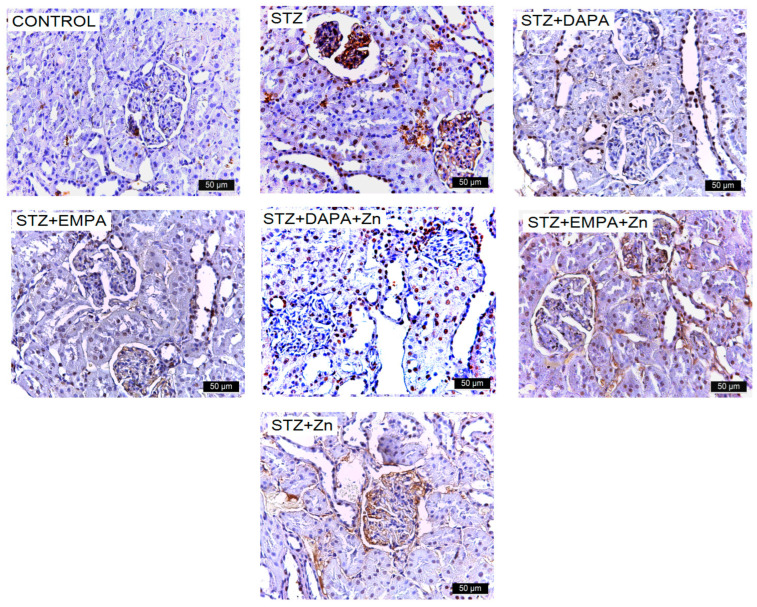
Representative immunohistochemical images of ICAM-1 expression in kidney tissue of rats with STZ-induced T2DM. Kidney sections were collected from the cortical region at the end of the experimental period (T2). Images are representative of findings observed in all animals from each experimental group (n = 5 per group). Increased immunoreactivity for ICAM-1 is observed in the STZ group. Treatment with DAPA and EMPA, alone or in combination with Zn, reduced marker expression, with the most pronounced effect observed in the EMPA + Zn group. All images are presented at the same magnification (×400), and scale bars represent 50 μm. STZ: streptozotocin; DAPA: dapagliflozin; EMPA: empagliflozin. VCAM-1 expression in the kidneys was assessed across both control and experimental groups. The highest levels were observed in the STZ and STZ + Zn groups, particularly within the Malpighian corpuscles (glomeruli). In the other experimental groups, VCAM-1 was detectable but substantially lower (Figure 9).

Increased ICAM-1 expression was observed in the kidneys of rats that developed T2DM, suggesting activation of inflammatory pathways associated with diabetic renal injury (Figure 8). The number of ICAM-1-positive cells ranged from 469.87 ± 193.73 to 4447.62 ± 91.02, with statistically significant differences between the group means (F(6, 28) = 155.99, *p* < 0.001). The lowest mean value was recorded in the Control group (469.87 ICAM-1-positive cells/mm^2^ ± 193.73), while the highest mean level was observed in the STZ group (4447.62 ICAM-1-positive cells/mm^2^ ± 91.02) indicating impaired renal function following diabetes induction. Treatment with SGLT2 inhibitors attenuated this increase suggesting improvement in renal function following pharmacological intervention. The highest level of ICAM-1 immunoreactivity was observed among the treated groups in the STZ + DAPA group (3125.51 ICAM-1-positive cells/mm^2^ ± 104.19). However, the values remained significantly lower than in the untreated STZ group (*p* < 0.001) and showed no significant difference compared to the STZ + Zn group (2676.52 ICAM-1-positive cells/mm^2^ ± 342.85, *p* = 0.104) (Figure 7b).

The comparison between the control and STZ groups revealed a very large effect size (Cohen’s d ≈ 26.28), indicating a indicate an inflammatory effect associated with STZ-induced diabetes. Comparisons between the STZ group and treated groups showed very large effect sizes, ranging from 5.97 to 26.45. The largest effect size was observed for the STZ vs. STZ + DAPA + Zn comparison (d ≈ 26.45) and for the STZ vs. STZ + EMPA + Zn comparison (d ≈ 23.35), suggesting a potential influence of combined therapy on ICAM-1 immunoreactivity.

**Figure 9:**
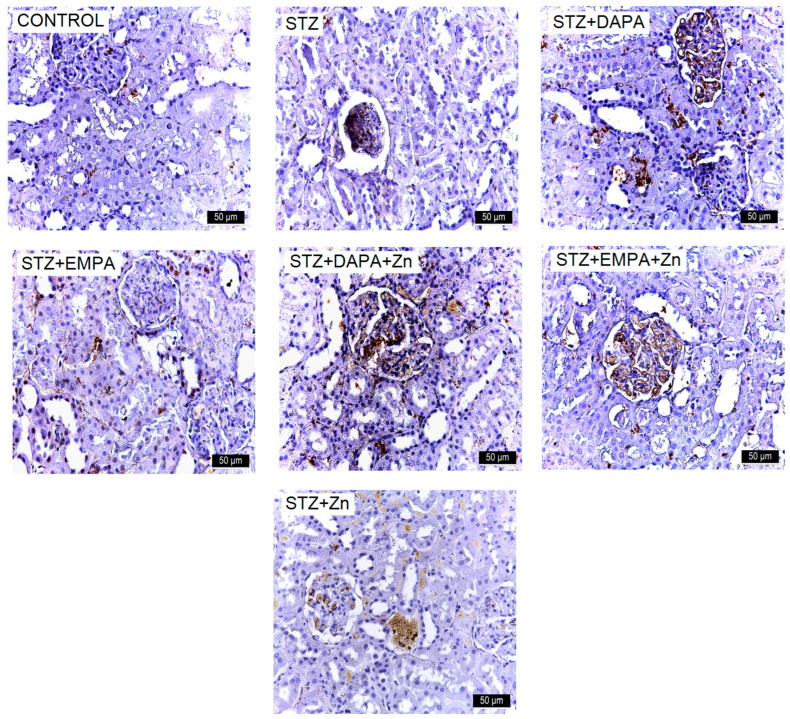
Representative immunohistochemical images of VCAM-1 expression in kidney tissue of rats with STZ-induced T2DM. Kidney sections were collected from the cortical region at the end of the experimental period (T2). Images are representative of findings observed in all animals from each experimental group (n = 5 per group). Increased immunoreactivity for VCAM-1 is observed in the STZ group. Treatment with DAPA and EMPA, alone or in combination with Zn, reduced marker expression, with the most pronounced effect observed in the EMPA + Zn group. All images are presented at the same magnification (×400), and scale bars represent 50 μm. STZ: streptozotocin; DAPA: dapagliflozin; EMPA: empagliflozin.

The number of VCAM-1-positive cells ranged from 512.82 ± 284.56 to 3150.47 ± 268.27, with statistically significant differences between the group means (F(6, 28) = 61.62, *p* < 0.001). The lowest mean value was found in the control group (512.82 VCAM-1-positive cells/mm^2^ ± 284.56), whereas the highest value occurred in the STZ group (3150.47 VCAM-1-positive cells/mm^2^ ± 268.27), confirming the presence of diabetes-associated renal impairment. This pattern indicates that VCAM-1 upregulation is closely associated with the STZ-induced diabetic state, reflecting increased inflammatory and adhesion-related activity in the glomeruli. Among the treated groups, the highest mean VCAM-1 immunolabeling was observed in the STZ + DAPA group (3046.03 VCAM-1-positive cells/mm^2^ ± 268.28), with no significant differences compared to the STZ + Zn group (2644.32 VCAM-1-positive cells/mm^2^ ± 589.58, *p* = 0.443) and the STZ + EMPA group (2587.83 ICAM-1-positive cells/mm^2^ ± 381.10, *p* = 0.294). The lowest level of VCAM-1 immunoreactivity was observed among the treated groups in the STZ + EMPA + Zn (811.27 VCAM-1-positive cells/mm^2^ ± 142.31) and STZ + DAPA + Zn groups (1139.68 VCAM-1-positive cells/mm^2^ ± 197.81). There were no significant differences between these groups (*p* = 0.668), indicating a strong renal protective effect of these combinations. Effect size analysis confirmed the magnitude of the differences in VCAM-1 immunolabeling levels among the experimental groups (Figure 7c). The comparison between the control and STZ groups showed an large effect size (Cohen’s d ≈ 9.54), reflecting the strong impact of STZ-induced diabetes on renal function. All treatment groups showed moderate to large reductions in VCAM-1 immunostaining levels compared with the STZ group, with Cohen’s d values ranging from 0.52 to 10.89, and the largest effect size observed for the STZ vs. STZ + EMPA + Zn comparison (d ≈ 10.89), indicating a strong renal protective effect of this association.

**Figure 10:**
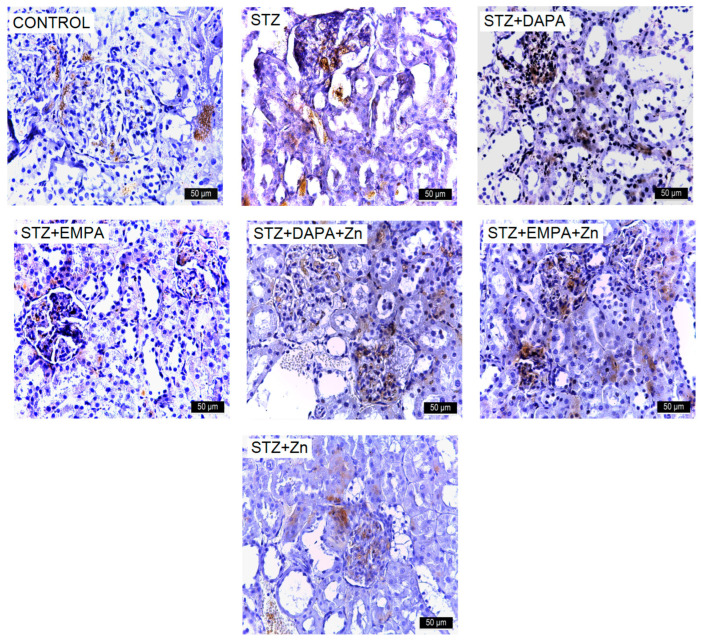
Representative immunohistochemical images of N-cadherin expression in kidney tissue of rats with STZ-induced T2DM. Kidney sections were collected from the cortical region at the end of the experimental period (T2). Images are representative of findings observed in all animals from each experimental group (n = 5 per group). Increased immunoreactivity for N-cadherin is observed in the STZ group. Treatment with DAPA and EMPA, alone or in combination with Zn, reduced marker expression, with the most pronounced effect observed in the EMPA + Zn group. All images are presented at the same magnification (×400), and scale bars represent 50 μm. STZ: streptozotocin; DAPA: dapagliflozin; EMPA: empagliflozin.

N-cadherin expression demonstrated positive immunostaining in the experimental groups. The number of N-cadherin-positive cells ranged from 457.13 ± 178.26 to 3048.43 ± 506.31, with statistically significant differences between the group means (F(6, 28) = 49.95, *p* < 0.001). The STZ group (3048.43 N-cadherin-positive cells/mm^2^ ± 506.31) exhibited higher N-cadherin immunoreactivity (*p* < 0.001) compared to the Control group (457.13 N-cadherin-positive cells/mm^2^ ± 178.26), confirming the presence of diabetes-associated renal impairment (Figure 7e). Pronounced degenerative morphological changes were observed at the glomerular level in the STZ group (Figure 10).

Treatment with SGLT2 inhibitors attenuated this increase. The STZ + EMPA + Zn group (448.30 N-cadherin-positive cells/mm^2^ ± 92.26) showed the lowest level of immunoreactivity among the treatment groups, with no significant differences (*p* = 0.36) compared to the STZ + DAPA + Zn group (901.43 N-cadherin-positive cells/mm^2^ ± 291.11) and the Control group (*p* > 0.05). The STZ + DAPA group (2575.85 N-cadherin-positive cells/mm^2^ ± 582.08) showed the highest number of N-cadherin-positive cells among the treatment groups, with no significant difference compared to the STZ + Zn group (2077.69 N-cadherin-positive cells/mm^2^ ± 109.94, *p* = 0.257) and STZ + EMPA group (2214.25 N-cadherin-positive cells/mm^2^ ± 233.82, *p* = 0.618). Effect size analysis confirmed the magnitude of the differences in N-cadherin immunostaining levels among the experimental groups. The comparison between the control and STZ groups showed an large effect size (Cohen’s d ≈ 6.83), reflecting the strong impact of STZ-induced diabetes on renal function. All treatment groups showed very large reductions in N-cadherin immunolabeling levels compared with the STZ group, with Cohen’s d values ranging from 0.84 to 7.14, and the largest effect size observed for the STZ vs. STZ + EMPA + Zn comparison (d ≈ 7.14), where STZ-induced renal lesions were appreciably ameliorated by this association.

**Figure 11:**
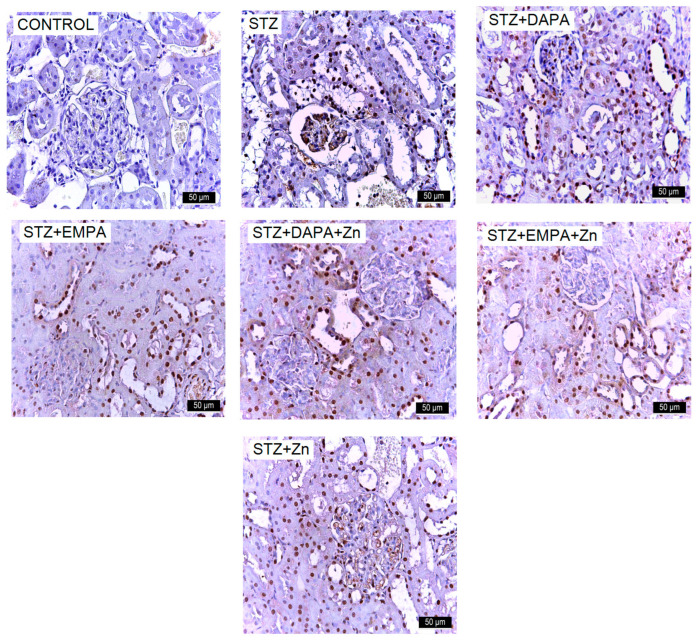
Representative immunohistochemical images of NF-κB expression in kidney tissue of rats with STZ-induced T2DM. Kidney sections were collected from the cortical region at the end of the experimental period (T2). Images are representative of findings observed in all animals from each experimental group (n = 5 per group). Increased immunoreactivity for NF-κB is observed in the STZ group. Treatment with DAPA and EMPA, alone or in combination with Zn, reduced marker expression, with the most pronounced effect observed in the EMPA + Zn group. All images are presented at the same magnification (×400), and scale bars represent 50 μm. STZ: streptozotocin; DAPA: dapagliflozin; EMPA: empagliflozin.

NF-κB expression was examined in the kidneys of both control and experimental groups (Figure 11). The number of NF-κB-positive cells ranged from 451.18 ± 113.26 to 3014.55 ± 153.25, with statistically significant differences between the group means (F(6, 28) = 64.11, *p* < 0.001). The lowest mean value was found in the control group (451.18 NF-κB-positive cells/mm^2^ ± 113.26), whereas the highest value occurred in the STZ group (3014.55 NF-κB-positive cells/mm^2^ ± 153.25), confirming the presence of diabetes-associated renal impairment (Figure 7d). These findings indicate that NF-κB activation is strongly associated with STZ-induced diabetic conditions, especially in the glomeruli, which are highly sensitive to inflammatory and oxidative stress.

Among the treated groups, the highest mean NF-κB immunolabeling was observed in the STZ + DAPA group (2830.82 NF-κB-positive cells/mm^2^ ± 213.75), with no significant differences compared to the STZ + Zn group (2659.82 NF-κB-positive cells/mm^2^ ± 538.22, *p* = 0.969) and the STZ + EMPA group (2588.35 NF-κB-positive cells/mm^2^ ± 422.47, *p* = 0.856). The lowest level of NF-κB immunoreactivity was observed among the treated groups in the STZ + EMPA + Zn (904.04 NF-κB-positive cells/mm^2^ ± 221.81) and STZ + DAPA + Zn groups (1074 NF-κB-positive cells/mm^2^ ± 175.55), with no significant differences between these groups (*p* = 0.907). There was no significant difference between the control group and the STZ + EMPA + Zn group also (*p* = 0.241). This suggests that lower, tubule-specific expression represents the basal level of NF-κB activity required for normal renal cellular function, rather than pathological activation.

The comparison between the control and STZ groups revealed a very large effect size (Cohen’s d ≈ 19.02), indicating a strong metabolic alteration associated with STZ-induced diabetes. Comparisons between the STZ group and treated groups showed moderate to large effect sizes, ranging from 0.90 to 11.78. The largest effect size was observed for the STZ vs. STZ + DAPA + Zn comparison (d ≈ 11.78) and for the STZ vs. STZ + EMPA + Zn comparison (d ≈ 11.07), suggesting a potential influence of combined therapy on NF-κB immunolabeling.

## 4. Discussion

The metabolic alterations observed in the STZ-treated animals are consistent with the well-established diabetogenic effects of STZ on pancreatic β-cells. Early experimental studies demonstrated that STZ selectively induces β-cell destruction, leading to persistent hyperglycemia and metabolic dysregulation [38]. Subsequent investigations confirmed that the use of a HFD in combination with low-dose STZ results in a metabolic state that closely mimics T3SM, characterized by insulin resistance and partial β-cell dysfunction [25,26]. The persistent hyperglycemia and altered body weight patterns observed in our study are therefore consistent with the metabolic profile described in these experimental models.

Treatment with the SGLT-2 inhibitors DAPA and EMPA produced measurable improvements in metabolic parameters, supporting the known physiological actions of this drug class. SGLT-2 inhibitors lower blood glucose levels mainly through the inhibition of glucose reuptake in the proximal renal tubules, thereby promoting glucosuria and improving glycemic control [38,39,40]. In addition to their glucose-lowering effects, these agents influence energy balance, body weight regulation, and renal hemodynamics, contributing to improved metabolic homeostasis [41]. Large clinical trials have confirmed the systemic benefits of these drugs. For example, the EMPA-REG OUTCOME trial demonstrated that EMPA notably reduces cardiovascular and renal events in diabetic patients [42], while the DECLARE–TIMI 58 trial reported similar cardiometabolic benefits for DAPA [43]. More recent analyses have further highlighted that SGLT-2 inhibitors improve metabolic regulation while reducing renal stress in both clinical and experimental settings [13,14].

In the present study, renal function was evaluated using serum creatinine, urea, and uric acid levels, which reflect glomerular filtration and nitrogen waste clearance. The biochemical alterations detected in the STZ group, including elevated serum creatinine and urea levels, reflect early renal dysfunction typical of diabetic nephropathy. Hyperglycemia-induced oxidative stress and glomerular hyperfiltration are recognized contributors to progressive renal injury in diabetes [6]. These pathological processes are associated with structural changes in glomeruli and renal tubules, as described in the pathological classification of diabetic nephropathy proposed by the Renal Pathology Society [44]. In the present study, treatment with SGLT-2 inhibitors partially improved these biochemical parameters, supporting their established renal protective effectsEffect size analysis confirmed that these changes were associated with large to extremely large biological effects. Treatment with SGLT2 inhibitors, particularly DAPA, partially attenuated the increases in creatinine and urea, suggesting a mitigation of renal damage in the diabetic nephropathy experimental setting.

The improvement in renal biochemical markers observed in treated animals is consistent with the mechanisms proposed for SGLT-2 inhibitors. Experimental studies have shown that these agents reduce intraglomerular pressure and mitigate hyperfiltration-induced injury by restoring tubuloglomerular feedback [45]. Clinical trials have provided further support for these mechanisms. The CREDENCE trial demonstrated that canagliflozin markedly reduces the risk of kidney failure and cardiovascular events in patients with diabetic nephropathy [45], while the DAPA-CKD trial confirmed that DAPA delays the evolution of CKD regardless of baseline glycemic status [13]. Similarly, the EMPA-KIDNEY study reported substantial protection against renal function decline in patients treated with EMPA [14].

Elevated uric acid levels in diabetic animals have also been previously described and are thought to result from altered renal urate transport and increased oxidative stress [46]. Although SGLT-2 inhibitors are generally associated with reduced serum uric acid due to enhanced uricosuria [47], the relatively higher uric acid levels observed in the STZ + DAPA + Zn group in the present study may reflect complex metabolic interactions within the experimental model. These results align with previously published literature showing that SGLT2 inhibitors are typically associated with decreased serum uric acid concentrations. This effect is thought to result from increased urinary urate elimination, secondary to pharmacologically induced glycosuria. Most clinical studies and meta-analyses have demonstrated a significant decrease in serum uric acid, with no consistent evidence of a sustained increase, although transient elevations may occur in certain clinical situations, such as dehydration or acute changes in plasma volume [48].

Histopathological evaluation of kidney specimens further confirmed the deleterious effects of STZ-induced diabetes on renal structure. The extensive glomerular and tubular damage observed in untreated diabetic animals is consistent with classical histopathological features of diabetic nephropathy, such as mesangial matrix expansion, thickening of the glomerular basement membrane, and progressive fibrosis of the tubulointerstitial compartment [6]. These alterations are largely driven by hyperglycemia-induced oxidative stress and inflammatory signaling pathways [22]. In our study, treatment with SGLT-2 inhibitors attenuated these structural lesions, supporting previous evidence that these agents can reduce renal inflammation and fibrosis while preserving tissue architecture [17,49,50].

The protective effects observed with Zn supplementation are also supported by previous studies highlighting the role of Zn in metabolic regulation and antioxidant defense. Zn plays an essential role in insulin synthesis, storage, and secretion and contributes to maintaining pancreatic β-cell integrity [51]. Additionally, Zn participates in the regulation of oxidative stress and inflammatory pathways that are critically involved in the pathogenesis of diabetic complications. Experimental evidence suggests that Zn supplementation can reduce oxidative damage and improve metabolic control in diabetic models [20].

The increased expression of mesenchymal markers observed in diabetic kidneys is consistent with activation of EMT, a process recognized as a key contributor to renal fibrosis. EMT involves the transdifferentiation of tubular epithelial cells into mesenchymal-like cells, leading to increased extracellular matrix deposition and progressive tissue remodeling [52]. Hyperglycemia-induced activation of TGF-β signaling pathways has been identified as a major driver of this process in diabetic nephropathy [8,53,54].

The reduction in α-SMA expression observed in animals treated with SGLT-2 inhibitors suggests that these agents may attenuate EMT-associated fibrotic signaling. Previous studies have shown that SGLT-2 inhibition suppresses renal inflammation and fibrosis through modulation of pathways such as NF-κB and TGF-β signaling [49,50]. These mechanisms are probably implicated in maintaining the structural integrity of renal tissue.

Although N-cadherin is not considered a classical biomarker of DKD, its inclusion in the present study was motivated by its important role in epithelial cell integrity and EMT, processes that are progressively acknowledged as playing a role in the development of renal fibrosis and tubular damage [55,56]. N-cadherin is a calcium-dependent cell adhesion molecule involved in maintaining intercellular junctions and structural stability within epithelial tissues. Alterations in cadherin-mediated cell adhesion have been associated with pathological remodeling in several renal diseases [57].

In the context of diabetic kidney injury, chronic hyperglycemia and oxidative stress can induce epithelial cell dysfunction and promote EMT-like processes in renal tubular cells. During EMT, epithelial cells undergo a gradual loss of their inherent polarity together with a reduction in cell–cell adhesion capabilities, while acquiring mesenchymal features that contribute to extracellular matrix deposition and fibrotic remodeling [52,58]. Although N-cadherin loss is traditionally considered a hallmark of EMT, increased or altered expression of N-cadherin has also been reported in association with epithelial phenotypic changes and tissue remodeling in renal pathology [59]. Furthermore, N-cadherin expression has been described in renal tubular epithelial cells and may reflect adaptive or pathological responses to cellular stress and injury. Several experimental studies have suggested that changes in N-cadherin expression may occur in conditions involving tubular damage, inflammation, and fibrotic processes, which are key features of diabetic nephropathy progression [60].

Therefore, the evaluation of N-cadherin in this study was intended to provide additional insight into potential alterations in epithelial cell adhesion and EMT-related processes associated with diabetic renal injury. By analyzing N-cadherin expression alongside established inflammatory and profibrotic indicators, including: NF-κB, ICAM-1, VCAM-1, and α-SMA, the study aimed to obtain a broader overview of the structural and molecular changes occurring in diabetic kidney tissue.

Finally, the increased expression of ICAM-1, VCAM-1, and NF-κB observed in STZ-treated animals highlights the key importance of inflammation in the development of diabetic nephropathy [9]. Hyperglycemia promotes endothelial activation and leukocyte recruitment through the upregulation of adhesion molecules and inflammatory cytokines [61,62,63]. In this context, the reduced expression of these markers following treatment with SGLT-2 inhibitors and Zn suggests a beneficial modulation of inflammatory pathways. Recent studies have similarly reported that SGLT-2 inhibitors exert anti-inflammatory actions by attenuating the redox imbalance and inhibiting the inflammatory signaling pathways in renal tissues [64,65,66].

The quantitative analysis of immunohistochemical markers performed in the present study provides additional support for the observed histopathological findings and strengthens the interpretation of the underlying mechanisms. By using digital image analysis to assess the number of positively stained cells per mm^2^, we were able to objectively evaluate the expression of key inflammatory and fibrotic markers, including α-SMA, ICAM-1, VCAM-1, NF-κB, and N-cadherin.

The results demonstrate that experimental diabetes was associated with a significant increase in the expression of these markers, reflecting activation of pro-inflammatory and pro-fibrotic pathways. Treatment with SGLT2 inhibitors, particularly when combined with zinc supplementation, resulted in a marked reduction in immunopositivity, suggesting attenuation of these pathological processes. These findings are consistent with the known anti-inflammatory and antifibrotic effects of SGLT2 inhibition and support the hypothesis that zinc may exert complementary protective effects, possibly through modulation of oxidative stress and inflammatory signaling pathways.

Importantly, the integration of semi-quantitative immunohistochemical analysis with biochemical and histological data enhances the overall robustness of the study and provides a more comprehensive understanding of the mechanisms underlying renal protection in this experimental model. These findings support the concept that diabetic nephropathy results from the interplay of metabolic dysregulation, oxidative stress, inflammation, and fibrotic remodeling. The protective effects on renal tissue observed with SGLT-2 inhibitors in our experimental model are consistent with the growing body of clinical and experimental evidence demonstrating their capacity to preserve renal function. Zn supplementation may provide additional benefits through antioxidant and β-cell-protective mechanisms, suggesting that combined therapeutic strategies targeting multiple pathogenic pathways could enhance protection against diabetes-induced renal injury.

## 5. Limitations

Several limitations of this study should be acknowledged when interpreting the results. Initially, the experiment was conducted exclusively on male Sprague-Dawley rats, which may restrict the external validity of the findings to females or other strains, as sex and genetic background can influence both the development of T2DM and the response to pharmacological interventions. Second, the study focused on short- to medium-term outcomes, and long-term effects of SGLT-2 inhibitors, Zn supplementation, or their combination on renal function and metabolic parameters remain unclear.

Additionally, while multiple biochemical and histological markers were assessed, functional measures such as glomerular filtration rate or urinary albumin excretion were not comprehensively evaluated, which may limit the ability to fully characterize renal function. Despite this limitation, the biochemical markers used in this study are widely applied in preclinical models and provide relevant insight into renal involvement in the context of metabolic disease. Future studies should incorporate these functional endpoints to further validate the renoprotective effects observed.

Finally, the study relied on a STZ-induced model of T2DM, which, although widely used, may not completely replicate the complexity of human diabetic nephropathy, including interactions with comorbidities and environmental factors. These limitations suggest that further studies, including diverse animal models and longer-term follow-up periods, are required to confirm and further expand upon these findings.

## 6. Future Perspectives and Clinical Implications

The results of this study provide multiple directions for future research and have potential clinical implications. The demonstrated reno-protective effects of SGLT-2 inhibitors, alone or in association with Zn, suggest that targeting both metabolic control and oxidative or inflammatory pathways could be a promising strategy for preventing or delaying the advancement of diabetic nephropathy. Future research is warranted to assess the long-term efficacy and safety profile of these agents, including their effects on glomerular filtration, proteinuria, and cardiovascular outcomes, as well as potential sex- and age-related differences in response. Additionally, mechanistic investigations into the modulation of EMT, inflammatory signaling, and oxidative stress pathways may help identify biomarkers for early-stage diagnosis and monitoring of renal impairment in diabetic patients. Clinically, these findings support the potential for combined therapeutic approaches that go beyond glucose lowering, emphasizing renal protection as a key component of T2DM management and offering insights for personalized treatment strategies aimed at reducing the burden of DKD.

The translational implications of this research are important for the management and prevention of diabetic nephropathy in humans. First, the data emphasize the possible clinical utility of SGLT-2 inhibitors, such as DAPA and EMPA, not only as glucose-lowering agents but also as reno-protective therapies capable of reducing inflammation, fibrosis, and EMT in the kidney. Second, the enhanced effect observed with Zn supplementation suggests that adjunctive nutritional or pharmacological strategies targeting oxidative stress and β-cell support may further improve renal outcomes in diabetic patients. Third, the study identifies molecular markers, such as ICAM-1, VCAM-1, and α-SMA, as potential early indicators of renal injury, which could guide patient monitoring and risk stratification in clinical practice. Finally, these results highlight the importance of combination therapies that address both metabolic dysregulation and renal tissue remodeling, providing a rationale for future clinical trials aimed at preventing the progression of DKD and improving both kidney function and cardiovascular function in individuals with T2DM over the long term.

## 7. Conclusions

The present study demonstrates that STZ-induced diabetes results in marked metabolic, biochemical, and structural alterations characteristic of diabetic nephropathy. Sustained hyperglycemia was closely associated with impaired renal function, pronounced histopathological lesions, and upregulated expression of key inflammatory and fibrotic markers, including NF-κB, ICAM-1, VCAM-1, α-SMA, and N-cadherin.

Administration of the SGLT2 inhibitors DAPA and EMPA led to significant improvements in metabolic control and renal function parameters. These effects were accompanied by attenuation of renal structural damage and downregulation of major inflammatory and fibrotic mediators. Such findings are in line with accumulating evidence consistent with the renal protective activity of SGLT2 inhibitors in the context of DKD.

Notably, the addition of Zn supplementation further enhanced these therapeutic effects, indicating a potential synergistic interaction between pharmacological glucose-lowering strategies and micronutrient-mediated antioxidant defense. Zn may exert its beneficial role through modulation of oxidative stress, improvement of metabolic homeostasis, and preservation of pancreatic β-cell integrity.

Among the evaluated therapeutic regimens, the combination of EMPA and Zn exhibited superior protective effects, as reflected by reduced expression of inflammatory markers and improved preservation of renal histoarchitecture.

These results suggest that therapeutic approaches targeting both metabolic dysregulation and inflammatory–fibrotic pathways may offer superior protection against diabetes-induced renal injury. Further experimental and clinical investigations are warranted to elucidate the molecular mechanisms underlying the potential synergistic effects of SGLT2 inhibitors in combination with Zn in DKD.

## Data Availability

The original contributions presented in this study are included in the article. Further inquiries can be directed to the corresponding author.

## Supplementary Materials

The following supporting information can be downloaded at: https://www.mdpi.com/article/10.3390/life16050793/s1, Table S1: Effect size (Cohen’s d) and estimated statistical power for serum creatinine (n = 5, α = 0.05, comparisons made against the STZ group (diabetes mellitus model in rats); Table S2: Effect size (Cohen’s d) and estimated statistical power for serum urea (n = 5, α = 0.05, comparisons made against the STZ group (diabetes mellitus model in rats); Table S3. Effect size (Cohen’s d) and estimated statistical power for serum uric acid (n = 5, α = 0.05, comparisons made against the STZ group (diabetes mellitus model in rats).

## References

[1] Sun H., Saeedi P., Karuranga S., Pinkepank M., Ogurtsova K., Duncan B.B., Stein C., Basit A., Chan J.C.N., Mbanya J.C. (2022). IDF Diabetes Atlas: Global, Regional and Country-Level Diabetes Prevalence Estimates for 2021 and Projections for 2045. Diabetes Res. Clin. Pract..

[2] Saeedi P., Petersohn I., Salpea P., Malanda B., Karuranga S., Unwin N., Colagiuri S., Guariguata L., Motala A.A., Ogurtsova K. (2019). Global and Regional Diabetes Prevalence Estimates for 2019 and Projections for 2030 and 2045: Results from the International Diabetes Federation Diabetes Atlas, 9th Edition. Diabetes Res. Clin. Pract..

[3] Alicic R.Z., Rooney M.T., Tuttle K.R. (2017). Diabetic Kidney Disease: Challenges, Progress, and Possibilities. Clin. J. Am. Soc. Nephrol..

[4] Thomas M.C., Brownlee M., Susztak K., Sharma K., Jandeleit-Dahm K.A., Zoungas S., Rossing P., Groop P.H., Cooper M.E. (2019). Diabetic Kidney Disease. Nat. Rev. Dis. Prim..

[5] Tuttle K.R., Bakris G.L., Bilous R.W., Chiang J.L., De Boer I.H., Goldstein-Fuchs J., Hirsch I.B., Kalantar-Zadeh K., Narva A.S., Navaneethan S.D. (2014). Diabetic Kidney Disease: A Report from an ADA Consensus Conference. Diabetes Care.

[6] Forbes J.M., Cooper M.E. (2013). Mechanisms of Diabetic Complications. Physiol. Rev..

[7] Reidy K., Kang H.M., Hostetter T., Susztak K. (2014). Molecular Mechanisms of Diabetic Kidney Disease. J. Clin. Investig..

[8] Liu Y. (2011). Cellular and Molecular Mechanisms of Renal Fibrosis. Nat. Rev. Nephrol..

[9] Navarro-González J.F., Mora-Fernández C. (2008). The Role of Inflammatory Cytokines in Diabetic Nephropathy. J. Am. Soc. Nephrol..

[10] Kaur P., Dahiya R., Nandave M., Sharma K., Goyal R.K. (2024). Unveiling the crucial role of intercellular adhesion molecule-1 in secondary diabetic complications. Cell Biochem. Funct..

[11] Podestà M.A., Sabiu G., Galassi A., Ciceri P., Cozzolino M. (2023). SGLT2 Inhibitors in Diabetic and Non-Diabetic Chronic Kidney Disease. Biomedicines.

[12] Dharia A., Khan A., Sridhar V.S., Cherney D.Z.I. (2023). SGLT2 Inhibitors: The Sweet Success for Kidneys. Annu. Rev. Med..

[13] Heerspink H.J.L., Stefánsson B.V., Correa-Rotter R., Chertow G.M., Greene T., Hou F.F., Mann J.F.E., McMurray J.J.V., Lindberg M., Rossing P. (2020). Dapagliflozin in Patients with Chronic Kidney Disease. N. Engl. J. Med..

[14] Herrington W.G., Staplin N., Wanner C., Green J.B., Hauske S.J., Emberson J., Preiss D., Judge P.K., Mayne K.J., Ng S.Y.A. (2023). Empagliflozin in Patients with Chronic Kidney Disease. N. Engl. J. Med..

[15] Wanner C., Inzucchi S.E., Lachin J.M., Fitchett D., von Eynatten M., Mattheus M., Johansen O.E., Woerle H.J., Broedl U.C., Zinman B. (2016). Empagliflozin and Progression of Kidney Disease in Type 2 Diabetes. N. Engl. J. Med..

[16] Thomas M.C., Neuen B.L., Twigg S.M., Cooper M.E., Badve S.V. (2023). SGLT2 Inhibitors for Patients with Type 2 Diabetes and Chronic Kidney Disease: A Narrative Review. Endocr. Connect..

[17] Tartau C.G., Boboc I.K.S., Mititelu-Tartau L., Bogdan M., Buca B.R., Pavel L.L., Amalinei C. (2025). Exploring the protective effects of traditional antidiabetic medications and novel antihyperglycemic agents in diabetic rodent models. Pharmaceuticals.

[18] Olechnowicz J., Tinkov A., Skalny A., Suliburska J. (2018). Zinc status is associated with inflammation, oxidative stress, lipid, and glucose metabolism. J. Physiol. Sci..

[19] Chausmer A.B. (1998). Zinc, Insulin and Diabetes. J. Am. Coll. Nutr..

[20] Maret W. (2017). Zinc in Pancreatic Islet Biology, Insulin Sensitivity, and Diabetes. Prev. Nutr. Food Sci..

[21] Ranasinghe P., Pigera S., Galappatthy P., Katulanda P., Constantine G.R. (2015). Zinc and Diabetes Mellitus: Understanding Molecular Mechanisms and Clinical Implications. DARU J. Pharm. Sci..

[22] Wang Y., Chen X., Song Y., Caballero B., Cheskin L.J. (2008). Association between Obesity and Kidney Disease: A Systematic Review and Meta-Analysis. Kidney Int..

[23] Jha J.C., Banal C., Chow B.S., Cooper M.E., Jandeleit-Dahm K. (2016). Diabetes and Kidney Disease: Role of Oxidative Stress. Antioxid. Redox Signal..

[24] Anton I.C., Mititelu-Tartau L., Popa E.G., Poroch M., Poroch V., Pelin A.-M., Pavel L.L., Drochioi I., Botnariu G.E. (2022). Zinc chloride enhances the antioxidant status, improving the functional and structural organic disturbances in streptozotocin-induced diabetes in rats. Medicina.

[25] Anton I.C., Mititelu-Tartau L., Iliescu R., Șerban I.L., Hăncianu M., Mircea C.G. (2023). Zinc potentiates the antioxidant effect of dapagliflozin in rats with experimental-induced diabetes. Med. Surg. J..

[26] Srinivasan K., Viswanad B., Asrat L., Kaul C.L., Ramarao P. (2005). Combination of High-Fat Diet-Fed and Low-Dose Streptozotocin-Treated Rat: A Model for Type 2 Diabetes and Pharmacological Screening. Pharmacol. Res..

[27] Skovsø S. (2014). Modeling Type 2 Diabetes in Rats Using High-Fat Diet and Streptozotocin. J. Diabetes Investig..

[28] Sagoo M.K., Gnudi L. (2021). Diabetic Nephropathy: An Overview. Methods in Molecular Biology.

[29] Wahono A.M., Harnanik T., Pasaribu I.A., Adiwinoto R.P., Octavianda Y. (2023). Laboratory and clinical findings in mouse models of diabetic nephropathy induced with streptozotocin. BMC Endocr. Disord..

[30] Tramunt B., Smati S., Grandgeorge N., Lenfant F., Arnal J.F., Montagner A., Gourdy P. (2020). Sex differences in metabolic regulation and diabetes susceptibility. Diabetologia.

[31] Srinivasan K., Ramarao P. (2007). Animal models in type 2 diabetes research: An overview. Indian J. Med. Res..

[32] Lu X., Xie Q., Pan X., Zhang R., Zhang X., Peng G., Zhang Y., Sumin Shen S., Tong N. (2024). Type 2 diabetes mellitus in adults: Pathogenesis, prevention and therapy. Signal Transduct. Target. Ther..

[33] Singh R., Gholipourmalekabadi M., Shafikhani S.H. (2024). Animal models for type 1 and type 2 diabetes: Advantages and limitations. Front. Endocrinol..

[34] Lee G., Goosens K.A. (2015). Sampling Blood from the Lateral Tail Vein of the Rat. J. Vis. Exp..

[35] Harikrishnan V., Hansen A.K., Abelson K.S., Sørensen D.B. (2018). A comparison of various methods of blood sampling in mice and rats: Effects on animal welfare. Lab. Anim..

[36] European Parliament and Council Directive 2010/63/EU on the Protection of Animals Used for Scientific Purposes. EUR-Lex. https://eur-lex.europa.eu/eli/dir/2010/63/oj.

[37] Legea nr. 43/2014 Privind Protecția Animalelor Utilizate în Scopuri Științifice. https://legislatie.just.ro/Public/DetaliiDocument/52457.

[38] Ma C., Li X., Li W., Li Y., Shui F., Zhu P. (2023). The Efficacy and Safety of SGLT2 Inhibitors in Patients with Non-Diabetic Chronic Kidney Disease: A Systematic Review and Meta-Analysis. Int. Urol. Nephrol..

[39] Junod A., Lambert A.E., Stauffacher W., Renold A.E. (1969). Diabetogenic Action of Streptozotocin: Relationship of Dose to Metabolic Response. J. Clin. Investig..

[40] Vallon V. (2015). The Mechanisms and Therapeutic Potential of SGLT2 Inhibitors in Diabetes Mellitus. Annu. Rev. Med..

[41] Vallon V., Thomson S.C. (2017). Targeting Renal Glucose Reabsorption to Treat Hyperglycaemia: The Pleiotropic Effects of SGLT2 Inhibition. Diabetologia.

[42] Zinman B., Wanner C., Lachin J.M., Fitchett D., Bluhmki E., Hantel S., Mattheus M., Devins T., Johansen O.E., Woerle H.J. (2015). Empagliflozin, Cardiovascular Outcomes, and Mortality in Type 2 Diabetes. N. Engl. J. Med..

[43] Wiviott S.D., Raz I., Bonaca M.P., Mosenzon O., Kato E.T., Cahn A., Silverman M.G., Zelniker T.A., Kuder J.F., Murphy S.A. (2019). Dapagliflozin and Cardiovascular Outcomes in Type 2 Diabetes. N. Engl. J. Med..

[44] Tervaert T.W., Mooyaart A.L., Amann K., Cohen A.H., Cook H.T., Drachenberg C.B., Ferrario F., Fogo A.B., Haas M., de Heer E. (2010). Pathologic Classification of Diabetic Nephropathy. J. Am. Soc. Nephrol..

[45] Perkovic V., Jardine M.J., Neal B., Bompoint S., Heerspink H.J.L., Charytan D.M., Edwards R., Agarwal R., Bakris G., Bull S. (2019). Canagliflozin and Renal Outcomes in Type 2 Diabetes and Nephropathy. N. Engl. J. Med..

[46] Johnson R.J., Nakagawa T., Sanchez-Lozada L.G., Shafiu M., Sundaram S., Le M., Ishimoto T., Sautin Y.Y., Lanaspa M.A. (2013). Sugar, Uric Acid, and the Etiology of Diabetes and Obesity. Diabetes.

[47] Chino Y., Samukawa Y., Sakai S., Nakai Y., Yamaguchi J., Nakanishi T., Tamai I. (2014). SGLT2 Inhibitor Lowers Serum Uric Acid through Alteration of Uric Acid Transport Activity in Renal Tubule. Diabetes Obes. Metab..

[48] Zhao Y., Xu L., Tian D., Xia P., Zheng H., Wang L., Chen L. (2018). Effects of sodium-glucose co-transporter 2 (SGLT2) inhibitors on serum uric acid level: A meta-analysis of randomized controlled trials. Diabetes Obes. Metab..

[49] Terami N., Ogawa D., Tachibana H., Hatanaka T., Wada J., Nakatsuka A., Eguchi J., Horiguchi C.S., Nishii N., Yamada H. (2014). Long-Term Treatment with the SGLT2 Inhibitor Ameliorates Diabetic Nephropathy. PLoS ONE.

[50] Zhang Z., Li Y., Gu H.F., Li N. (2025). Pharmacological mechanisms and clinical applications between SGLT1 and SGLT2 inhibitors in type 2 diabetes and diabetic kidney disease: An analytic review. Biomed. Pharmacother..

[51] Klein E., Velina D., Mutallibzoda S., Tefikova S., Orlovtseva O., Kosenkov A.N., Kulikov D., Nikitin I. (2025). Zinc and Type 2 Diabetes: A Systematic Review with a Narrative Synthesis of Their Bidirectional Relationship and Clinical Perspectives for Personalized Nutritional Support. Diseases.

[52] Zeisberg M., Neilson E.G. (2009). Biomarkers for Epithelial–Mesenchymal Transitions. J. Clin. Investig..

[53] Hou G., Dong Y., Jiang Y., Zhao W., Zhou L., Cao S., Li W. (2025). Immune Inflammation and Metabolic Interactions in the Pathogenesis of Diabetic Nephropathy. Front. Endocrinol..

[54] Liu X., Zhang C., Fu Y., Xie L., Kong Y., Yang X. (2025). Inflammation, Apoptosis, and Fibrosis in Diabetic Nephropathy: Molecular Crosstalk in Proximal Tubular Epithelial Cells. Curr. Issues Mol. Biol..

[55] Wang Y., Jin M., Cheng C.K., Li Q. (2023). Tubular injury in diabetic kidney disease: Molecular mechanisms and potential therapeutic perspectives. Front. Endocrinol..

[56] Lv Y., Chen Y., Zixi L., Ye J., Cao H., Zhang C., Jiang H., Wang Y. (2025). Tubular injury in diabetic kidney disease: Early diagnosis and intervention strategies. Metab. Res. Rev..

[57] Hu Q., Saleem K., Pandey J., Charania A.N., Zhou Y., He C. (2023). Cell Adhesion Molecules in Fibrotic Diseases. Biomedicines.

[58] Liu Y. (2010). New insights into epithelial-mesenchymal transition in kidney fibrosis. J. Am. Soc. Nephrol..

[59] Ali A., Abu Farha N., Hammad M., Devarajan K., Bahbahani Y., Al Khairi I., Cherian P., Alsairafi Z., Vijayan V., Al-Mulla F. (2022). Potential Role of N-Cadherin in Diagnosis and Prognosis of Diabetic Nephropathy. Front. Endocrinol..

[60] Nürnberger J., Feldkamp T., Kavapurackal R., Opazo Saez A., Becker J., Hörbelt M., Kribben A. (2010). N-Cadherin Is Depleted from Proximal Tubules in Experimental and Human Acute Kidney Injury. Histochem. Cell Biol..

[61] Deng Y., Zhang S., Luo Z., He P., Ma X., Ma Y., Wang J., Zheng L., Tian N., Dong S. (2024). VCAM1: An Effective Diagnostic Marker Related to Immune Cell Infiltration in Diabetic Nephropathy. Front. Endocrinol..

[62] Wang Y., Cheng G., Wang G., Zhou X., Ma M., Wang J. (2025). Predictive and Diagnostic Value of MCP-1, MIF, and ICAM-1 in Type 2 Diabetes Mellitus Patients with Diabetic Kidney Disease. Pak. J. Med. Sci..

[63] Abhirami B.L., Krishna A.A., Kumaran A., Chiu C.H. (2025). Targeting NF-κB in diabetic nephropathy: Exploring the therapeutic potential of phytoconstituents. Arch. Pharmacal Res..

[64] Zhang R., Xie Q., Lu X., Fan R., Tong N. (2024). Research advances in the anti-inflammatory effects of SGLT inhibitors in type 2 diabetes mellitus. Diabetol. Metab. Syndr..

[65] Guerrero-Mauvecin J., Villar-Gómez N., Miño-Izquierdo L., Povo-Retana A., Ramos A.M., Ruiz-Hurtado G., Sanchez-Niño M.D., Ortiz A., Sanz A.B. (2025). Antioxidant Effects of SGLT2 Inhibitors on Cardiovascular–Kidney–Metabolic (CKM) Syndrome. Antioxidants.

[66] Hirashima Y., Nakano T., Torisu K., Aihara S., Wakisaka M., Kitazono T. (2024). SGLT2 inhibition mitigates transition from acute kidney injury to chronic kidney disease by suppressing ferroptosis. Sci. Rep..

