# Supplementary material for: Zinc Potentiates the Renoprotective Effects of SGLT2 Inhibitors in Experimental Diabetes Mellitus in Rats
Source: Life (Basel). 2026 May 9;16(5):793. doi: 10.3390/life16050793 (PMC13208674; doi:10.3390/life16050793)
Supplement: Supplementary file 1 [file life-16-00793-s001.zip › life-4266831-supplementary.pdf]

Table S1. Effect size (Cohen`s d) and estimated statistical power for serum creatinine (n = 5, α = 0.05, comparisons made against the STZ group (diabetes mellitus model in rats).

Group	Mean (mg/dL)	SD	Cohen's d vs STZ	Effect Size Interpretation	Estimated Power
Control (M)	0.42	0.02	14.8	Extremely large	>99%
STZ	0.79	0.03	—	Reference	—
STZ + DAPA	0.50	0.02	10.3	Extremely large	>99%
STZ + EMPA	0.52	0.04	8.5	Extremely large	>99%
STZ + DAPA + Zn	0.51	0.03	10.6	Extremely large	>99%
STZ + EMPA + Zn	0.52	0.06	7.1	Extremely large	>99%
STZ + Zn	0.57	0.03	7.3	Extremely large	>99%

Table S2. Effect size (Cohen`s d) and estimated statistical power for serum urea (n = 5, α = 0.05, comparisons made against the STZ group (diabetes mellitus model in rats).

Group	Mean (mg/dl)	SD	Cohen's d vs STZ	Effect Size Interpretation	Estimated Power
Control (M)	32.80	2.39	2.89	Extremely large	>99%
STZ	71.80	18.97	—	Reference	—
STZ + DAPA	45.80	4.87	1.84	Very large	~90%
STZ + EMPA	40.80	8.35	1.67	Very large	~85%
STZ + DAPA + Zn	44.80	4.38	1.90	Very large	~91%
STZ + EMPA + Zn	38.60	6.88	1.77	Very large	~88%
STZ + Zn	40.80	2.59	1.93	Very large	~92%

Table S3. Effect size (Cohen`s d) and estimated statistical power for serum uric acid (n = 5, α = 0.05, comparisons made against the STZ group (diabetes mellitus model in rats).

Group	Mean (mg/dl)	SD	Cohen's d vs STZ	Effect Size Interpretation	Estimated Power
Control (M)	0.83	0.12	4.06	Very large	~97%
STZ	1.34	0.13	—	Reference	—
STZ + DAPA	1.12	0.20	1.29	Large	~75%
STZ + EMPA	1.16	0.23	0.99	Moderate–large	~60%
STZ + DAPA + Zn	1.19	0.07	1.40	Large	~80%
STZ + EMPA + Zn	1.17	0.04	1.72	Very large	~90%
STZ + Zn	1.17	0.28	0.74	Moderate	~45%
